# The Role of Health Psychology in Surgical Prehabilitation: Insights From REST, a Preoperative Sleep Intervention for Total Knee Replacement Patients

**DOI:** 10.1002/msc.70088

**Published:** 2025-03-28

**Authors:** Katie Whale, Emma Johnson, Rachael Gooberman‐Hill

**Affiliations:** ^1^ NIHR Bristol Biomedical Research Centre Bristol UK; ^2^ Musculoskeletal Research Unit University of Bristol Medical School Bristol UK

**Keywords:** behaviour change, health psychology, joint replacement, orthopaedic, patient experience, prehabilitation, psychology

## Abstract

**Background:**

Approximately 10%–34% of people experience chronic pain after total knee replacement (TKR) surgery. Prehabilitation approaches that address pre‐operative risk factors for chronic post‐surgical pain are a key area for research. To be effective, prehabilitation requires substantial engagement and behaviour change by patients, which can be challenging in the pre‐operative period. Health psychology theory plays a valuable role in understanding how best to support behaviour change to achieve maximum patient benefit. This study provides insights from REST, a pre‐operative sleep intervention for TKR patients.

**Methods:**

In‐depth semi‐structured interviews were conducted with eight TKR patients who took part in the REST feasibility trial. An abductive analysis approach was used to identify the applicability of existing health psychology theories, and to explore new insights into the relationships between stages of behaviour change.

**Results:**

Three thematic areas related to intervention engagement and enactment were identified: (i) health beliefs and readiness to change; (ii) from contemplation to enactment: the role of behaviour change techniques; (iii) and behavioural maintenance.

**Conclusion:**

Findings highlighted three key stages of behaviour change that participants need to be supported in to benefit fully from prehabilitation intervention. Complex behaviour change interventions that include aspects of tailoring should consider the boundaries of acceptable adaption while maintaining core causal mechanisms, and include methods to explore real‐world implementation and usability during the development process. These findings are important for surgeons and multidisciplinary teams to consider when developing new prehabilitation care pathways or when implementing evidence‐based prehabilitation practices.

## 1 Introduction

Total knee replacement (TKR) is the second most common elective surgical procedure in the UK with approximately 100,000 performed yearly (Registry 2022). Internationally, > 1 million operations are performed annually, with rates increasing by 22% between 2009 and 2019 (OECD 2021). For many people, TKR is effective as it provides improvements in knee function and pain. However, approximately 10%–34% experience chronic post‐surgical pain (pain lasting for longer than 3‐months) (Beswick et al. 2012), leading to dissatisfaction and disappointment (Howells et al. 2016; Kim et al. 2009). People living with pain after surgery describe difficulties managing work and social life, negative emotional impact, and anxiety and fear around further treatment (Sellevold et al. 2023).

There is a need to develop new approaches to reduce the incidence and severity of chronic pain after TKR. Prehabilitation interventions (pre‐operative rehabilitation) that address pre‐operative risk factors for developing chronic post‐surgical pain are key areas of research need (Almeida et al. 2020; Khalid et al. 2024; Vervullens et al. 2023). The strongest predictor of pain after TKR is pre‐surgical pain (Lewis et al. 2015; Sayers et al. 2016; Wylde et al. 2011): high pre‐operative pain, pain in other bodily locations, and pain catastrophising are all predictive of greater post‐operative pain severity and increased likelihood of developing chronic post‐surgical pain (Sayers et al. 2016). Therefore, approaches that target pain before surgery may provide ways to reduce the proportion of people who will experience chronic post‐surgical pain and may reduce the severity for those who do find that they have such pain.

Sleep is a known vital component of pain experience: reduced sleep is associated with increased nociceptive and neuropathic pain responses (Finan et al. 2013; Haack et al. 2007), and improvements to sleep can reduce both immediate and long‐term pain in osteoarthritis (Vitiello et al. 2009). Sleep is a substantial issue for people awaiting TKR with an estimated 70% experiencing sleep problems (Hawker et al. 2010; Manning et al. 2017; Wilcox et al. 2000). People report problems with getting to sleep, staying asleep, and frequently night waking (M. T. Smith et al. 2009). People with poorer sleep have high levels of pain catastrophising, anxiety, and depression (Boye Larsen et al. 2021), all factors that predict worse post‐surgical outcomes (Jiang et al. 2017; Judge et al. 2012). Pre‐operative sleep problems impair healing, increase infection risk (Wright et al. 2015), and can extend hospital stays by 1–8 days (Ding et al. 2022). Patients with pre‐existing sleep problems are five times more likely to develop post‐operative delirium (Fadayomi et al. 2018).

Research on sleep and TKR has predominantly focused on pharmacological interventions in the peri‐ and post‐operative period (Gong et al. 2015; Kirksey et al. 2015; Krenk et al. 2014). NICE and EULAR guidance for insomnia and pain management advise against pharmacological therapy for long‐term sleep management, recommending behavioural approaches as first‐line treatment (Geenen et al. 2018; NICE 2021), which are more sustainable and cost‐effective, with lower risk of side effects (Koffel et al. 2019). There is a paucity of research on non‐pharmacological pre‐operative interventions for TKR patients; however, there is a solid evidence base for behavioural sleep interventions for chronic pain patients, which offers promise. Three of the most well‐recognised approaches are cognitive behavioural therapy for insomnia (CBT‐i), relaxation, and mindfulness. All these studies demonstrate effectiveness in improving sleep for people experiencing chronic pain (Okajima et al. 2011; Sun et al. 2013; Wang et al. 2018; Whale et al. 2022). CBT‐i comprises personalised programmes to explore and change beliefs and behaviours that affect the ability to sleep. This includes establishing a bedtime routine, sleep restriction, and limiting time spent in bed awake. CBT‐i can be delivered in person with a therapist or through automated delivery via websites and apps (eCBT‐i) (Zachariae et al. 2016). Relaxation and mindfulness are the most common sleep self‐help techniques, including progressive muscle relaxation, breathing exercises, and attention regulation (Amutio et al. 2018; McClusky et al. 1991; Sun et al. 2013). Behavioural sleep interventions are commonly used in conjunction with sleep hygiene education. Sleep hygiene consists of advice and practical information about how to achieve a good sleep environment, for example, removing electronic devices from the bedroom and having a bedtime routine. Sleep hygiene education offers useful tools for making simple changes but has limited effectiveness as a standalone treatment with best practice to combine it with other intervention approaches (Chung et al. 2018).

To improve sleep for TKR patients, we developed the REST intervention (Whale and Gooberman‐Hill 2022). REST is a pre‐operative sleep intervention for people undergoing TKR that brings together evidence‐based existing sleep interventions to provide tailored sleep plans, underpinned by health psychology theory. We have conducted a two‐centre feasibility randomised controlled trial, already published (Bertram et al. 2024), and this article presents the results of the embedded qualitative study that explored intervention engagement and enactment.

Crucially, our study makes use of health psychology theory. Such theories are vital to the design and refinement of interventions that address health behaviours. Inclusion of theory is recommended in the Medical Research Council (MRC) guidance for intervention development (Craig et al. 2008; Skivington et al. 2021), with evidence that imbedding behaviour change theory increases intervention effectiveness (Webb et al. 2010). Understanding intervention engagement is a crucial part of intervention development and testing (Craig et al. 2008; Skivington et al. 2021). Successful implementation depends on the recipients being ready and willing to make changes (engagement), and being able to maintain these changes over time in order to experience benefit (enactment) (Sekhon et al. 2017). Health psychology theory provides important insight on how to support individuals through this process, and where barriers may occur. For example, the transtheoretical model aids understanding of an individual's readiness to act on a new health behaviour, and the process of moving from contemplation to action and maintenance. Theories of motivation, such as Self‐Determination Theory, provide insight into how change can be sustained, showing that interventions that support people's competency and autonomy lead to greater engagement, motivation, and long‐term behaviour change (Deci and Ryan 2008; O'Cathain et al. 2019; Ryan et al. 2008).

This article describes a qualitative research study, focussing on findings relating to engagement and enactment in the REST intervention.

## 2 The REST Intervention

REST is a tailored intervention delivered approximately 3 months pre‐surgery. Patients attend a remote one‐to‐one appointment with a health professional, lasting approximately 1 hour. In the appointment, a sleep assessment identifies individual sleep issues and needs. Based on the assessment, the professional works with the participant to provide sleep hygiene advice and recommends one of three existing sleep interventions (ESIs):

1. Cognitive behavioural therapy for insomnia is delivered through Sleepstation (an online platform provided by the UK's National Health Service: NHS).
2. Relaxation, delivered through the Calm app, guided audio and/or workbook.
3. Mindfulness meditation, delivered through the Headspace app, guided audio and/or workbook.

Participants are provided with a personalised sleep plan including SMART sleep hygiene goals, a detailed overview of their chosen ESI, and any materials. Participants receive a follow‐up call from the health professional 4 weeks after their appointment to review progress and engagement. This includes addressing barriers, reviewing of the sleep goals, and adjusting the sleep plan if needed.

## 3 Methods

### 3.1 Aims and Objectives

A qualitative interview study with study participants was conducted to assess the acceptability of REST. The secondary aims were to explore levels of engagement and enactment with the sleep plan and identify any areas for refinement. The qualitative study was embedded in a feasibility randomised trial. Feasibility trials are designed to ascertain whether and how a fully powered trial could happen (Eldridge et al. 2016). The REST feasibility trial found that it would be feasible to carry out a fully‐powered randomised controlled trial to evaluate the clinical and cost‐effectiveness of REST (Bertram et al. 2024). The qualitative study described here provides distinct information that can be used to refine and enhance the intervention itself.

### 3.2 Institutional Review Board Approval

This study received a favourable opinion and approval from the Health Research Authority and Health and Care Research (University of Bristol Research Data Storage Facility).

### 3.3 Participant Eligibility and Recruitment

Participants were eligible if they participated in the feasibility trial and were able to participate in an interview by telephone or videoconference. Trial participants were asked to complete a consent form, including a check box to indicate interest in the qualitative study. All individuals who indicated their interest in taking part were sent an invitation letter and reply slip and were contacted by the researcher. All participants provided written informed consent for the qualitative study.

### 3.4 Sampling Methods and Sample Size

Maximum variation sampling was used with sampling criteria of gender, age, and trial arm allocation. We aimed to include 20 intervention participants (10 per site) and 5 usual care participants. During the study and to maximise recruitment, qualitative sampling was expanded to include all intervention participants who had agreed to be contacted. This was needed due to the lower than anticipated number of feasibility trial participants due to the COVID‐19 pandemic reducing the overall number of elective surgical procedures. The total sample size was 13 participants, 8 intervention and 5 usual care. The intervention arm sample was smaller than originally planned. However, qualitative research does not always need to meet a pre‐defined or a priori sample size when information power is assessed (Malterud et al. 2016). Assessment of information power showed that the data in the sample enabled the study to meet its aims because of the tightly defined aims and rich data collected.

### 3.5 Data Collection

In‐depth semi‐structured interviews were conducted by an experienced qualitative researcher (EJ), informed by a topic guide developed in collaboration with the study's Patient and Public Involvement and Engagement (PPIE) group and on the basis of previous research in this area. Interviews explored experiences and acceptability of the REST intervention, engaging with the chosen ESI, and the impact of REST on sleep and pain. Interviews also elicited views about trial processes, which are presented in the article that reports trial results (Bertram et al. 2024).

Interviews were conducted either using videoconference or telephone, depending on the participants' preference. All interviews were recorded using an encrypted digital recorder.

### 3.6 Data Analysis

Data were analysed using an abductive approach (Vila‐Henninger et al. 2022). This was conducted separately from the analysis undertaken to address the feasibility research questions, as outlined in the trial paper. Abductive coding is a flexible approach that uses deductive and inductive analysis to support the exploration of existing theories and concepts, whilst still allowing for new insights to be identified from the data. One researcher (KW) read the transcripts and undertook two stages of data coding. Firstly, a deductive analysis identified concepts related to existing health psychology theory. Secondly, an inductive analysis identified additional data‐driven codes that did not align with existing theories or provided new insights into the relationship between stages of behaviour change. Analysis was conducted by one researcher (KW) with a background in health psychology. Codes were discussed with a second researcher (RGH) with a background in health and anthropology.

### 3.7 Patient and Public Involvement and Engagement

The study was developed in collaboration with a musculoskeletal PPIE group comprising 5 patients with lived experience of orthopaedic surgery. The group met four times during the research term to co‐design patient‐facing study materials, monitor study progress, and review results and dissemination plans.

## 4 Results

In total, 38 patients agreed to be contacted about taking part in an interview and were sent an invitation letter. The study team received reply slips from 16 participants, of whom 13 took part in an interview (*N* = 8 intervention/5 usual care). Of the remaining 3 after discussing the interview in more detail with the researcher (EJ), one decided that they were no longer interested in taking part and one declined due to a deterioration in health. The researcher was unsuccessful in contacting the third participant after multiple attempts. Interviews were between 24 and 86 min in duration and were conducted over the telephone (*N* = 5) or using a secure video platform (*N* = 8).

Intervention engagement was explored with only the eight intervention group participants. Demographics for the intervention group participants are presented in Table 1. All names are pseudonyms.

**TABLE 1:** Participant demographics.

Pseudonym	Gender	Age at interview	Ethnicity	Mode of intervention delivery	Chosen sleep intervention
Florence	Woman	90	Welsh	Telephone	Relaxation
Charles	Man	79	English	Video	CBT‐i
Patricia	Woman	69	White English	Video	CBT‐i
Gloria	Woman	73	White British	Video	Mindfulness
Ruth	Woman	72	English	Video	CBT‐i
Edward	Man	69	Welsh/British white	Telephone	Mindfulness
Jerry	Man	64	English	Video	Relaxation
Rose	Woman	68	White British	Video	Mindfulness then CBT‐i

## 5 Themes

**FIGURE 1:**
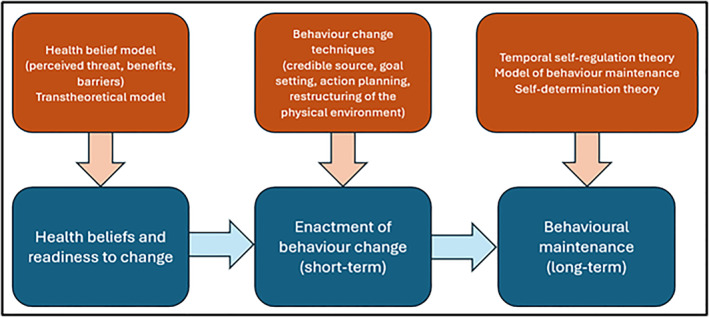
Diagram of behaviour change theory and processes.

Three thematic areas were identified: *Health beliefs and readiness to change; from contemplation to enactment: the role of behaviour change techniques; and Behavioural maintenance*. A diagram illustrating the relationship between each stage is presented in Figure 1. Illustrative quotations for each theme are presented in Table 2.

**TABLE 2:** Illustrative quotes.

Theme	Quote number	Quote	Participant
Health beliefs and readiness to change	1	“I would never go the full night. I Would maybe go four or 5 hours, then I'd wake up, then I'd got back to sleep again… So three or 4 hours, wake up, go to the toilet, walk around, take a tablet. Then I'd go back and sleep for another couple of hours. […] because it is a thing isn't it, sleep? You know, you really need it. If you can go through the night, some nights I can be up say two, three, four times and you do know if you've had a decent night sleep. So you do need the help that you're putting out.”	Jerry, age 64
	2	“ I mean compared to other people, there's only one person I know who sleeps worse than me and they're very neurotic [laughs] […]” I: What do you think kind of good sleep is? “… seven hours I think is fine for someone my age […] if it could be seven hours unbroken that’d be great. It’s very rare, maybe one in a hundred days I would say when I can sleep right through, wake up and it’s like, ‘I didn't wake up in the night.’ It's a very rare thing to do.”	Charles, age 79
	3	“…so I've always had trouble sleeping and pain and turning over in bed isn't good so when this trial came up about sleeping I thought this is absolutely perfect”	Rose, age 68
	4	“I don't think I've got an issue with sleep but everybody else does because I've never ever needed much sleep. […] So they're desperately at the moment trying to get me to do things that will mean I'll sleep for 6 hours, I'm never going to sleep for 6 hours […] I used to think it's quite blessed actually not wanting a lot of sleep because you don't waste, as I see it waste as much time… I can't see what harm it's done and it's given me a lot more life […] I'm fed up with this you know I don't have a sleep problem, you are the ones telling me I have a sleep problem.”	Ruth, age 72
	5	“I am deliberately waking up at 5 o'clock which is what they want me to do but not getting out of bed, laying in bed where it's warm, and I don't need the light on because I can switch on my little audio tape next to the bed … That's what I want to do and I am happy doing that, I do not want anybody to tell me not to do that, I don't see what's wrong, can you explain to me what's wrong with doing that.”—Ruth	Ruth, age 72
From contemplation to enactment: The role of behaviour change techniques	6	“Well I think I was speaking to a professional and when I feel that, that gives me a little confidence in talking to her… for me as being an amateur as it were and you being a professional, it gives me a little confidence in the way you are able to talk to me and I found that with [the practitioner] they were really very patient and in discussing their ideas of my walking and trying to walk more, they were very encouraging actually in what they was saying… I think they were gently pushing me on but in a very gentle way!”	Florence, age 90
	7	“It was good that I could say to her, ‘Well look [practitioner], did you mean that or could I do that?’, ‘No, no that's a good idea.’ You know what I mean? To feed off people, bounce of people and I could with her as well. She was very good.”	Ruth, age 72
	8	“I felt it was a nice in‐depth discussion and planning what I was going to do and how to improve my present circumstances, you know, with a way to relax and all those other suggestions she was making to me and my acceptance of whichever one I wanted to do, she made it very easy for me to make my own decisions.”	Florence, age 90
	9	“One thing that did come out and it really shocked me and that was how much coffee I was drinking because you know I talked very loosely over lots and lots of things and she came up with a great plan actually, it was no coffee after 12 o'clock midday and I've really stuck to that, I have really stuck to that because I didn't realise I was topping myself up all the time”	Rose, age 68
	10	“I went and got the decaffeinated drinks for everything and stuck to that and it's never been a problem. I have a fruit tea at tea time she did say I could have one in the afternoon because I always have a drink at 3 o'clock in the afternoon, but sometimes I do have a decaffeinated drink then but other times I will just have either water or fruit tea or something and it's really made a difference. I can recommend that highly enough actually.”	Rose, age 68
	11	“That is really helpful [having a sleep plan], all that sort of thing. Because I've got the time in the day, a certain amount of time, so I'm okay on that. I'm sitting upstairs and I'm sorting out say records up there, then I've got it on my file up there. And you do, you refer to it.”	Jerry, age 64
	12	“…not having the television on and being tempted to put the television on in the middle of the night, because sometimes I would watch television all night … so that is completely cut out of my life now … and that was really difficult because I used to go to bed, as I said, to rest my leg and I'd stopped doing that so now what I do is put both legs up on the sofa”	Rose, age 68
	13	“I read in bed, I mean he said do you turn off the television? I got rid of the television, don't have a television in there.”	Jerry, age 64
	14	“The other things was that we would always leave the toilet light on, so we could get up in the night and go to the loo and have some light, to which I've done two things with that. One is that I found on, well, both of them were on Amazon. I think, really that there's a little light that you can stick into a toilet bowl.”	Edward, age 69
	15	“[The practitioner] did suggest that I bought blackout linings for the curtains. So within 36 h of him saying that blackout linings were up and I think yes they have helped.”	Patricia, age 69
Behavioural maintenance	16	“I used to wake up just feeling I haven't been to bed at all. But now when I wake up, I think two nights ago I think that was the longest I've ever had. I Went to bed about 12, it might've been a bit later, and I woke up at quarter to six. That is unknown. And I can't remember whether I got up in the night at all. I may have done to go to the toilet, but I don't remember it and that was wonderful. And the difference it makes to your head and your wellbeing is amazing.”	Gloria, age 73
	17	“Before the REST study thing I used to wake up and go to the loo. I Think that was because of the coffee because the coffee is a diuretic [mmm] so yeah so that's… [so you are not waking up to use the toilet as much?] no, not as much, no because I don't have any drinks a couple of hours before I go to bed anyway.”	Rose, age 68
	18	“You know, I've made the changes. I mean not a lot of changes really I suppose. I've been changing the pattern of when I go to bed, changing the pattern of when I eat and drink at night and you know, that's made quite a big difference.”	Gloria, age 73
	19	“Headspace was just too distracting with the [images] yeah yeah so I didn't find that good. The mindfulness, the wake up the body and breathe body balance, I thought that was quite good. The body scanning is actually brilliant as I said with Diane Winson.”	Rose, age 68
	20	“I wouldn't wear headphones in bed because of the disruption of wearing headphones. With my wife was sleeping next to me, she didn't want to hear that all the time, but what I found then worked was that I reverted back to listening to Classic FM on Alexa. I asked Alexa to give me some meditation music first, but that was on meditation things, but I found that was a bit daft and a bit airy‐fairy.”	Edward, age 69
	21	“So it's not that the interventions were wrong. The concepts of the interventions were right, but the way that I put them into practice appeared to work for me.”	Edward, age 69

### 5.1 Health beliefs and Readiness to Change

In health psychology, health beliefs refer to a person's attitudes, values, and knowledge about health and health conditions. Readiness to change is whether or not an individual has reached the point that they are motivated and able to take action. Sleep beliefs played a pivotal role in an individual's readiness to change. Generally, participants had engaged with REST as they felt they had sleep issues and wanted to improve this. Participants said their personal motivation and belief that their sleep needed improvement meant they were highly engaged and ready to try out new techniques (quotes 1, 2 and 3).

Conversely, some participants felt they did not have sleep issues or their view of ‘healthy sleep’ differed from the REST guidance. One participant said their motivation for engaging with the study was to help research and others (quote 4). They saw no personal benefit in enacting the recommended behaviour change.

This incongruence caused negative emotions, such as stress and frustration, and disengagement from the recommended sleep plan (quote 5). This was particularly evident in relation to the CBT‐i recommendation to reduce time awake in bed.

These findings align with the health belief model that highlights the role of perceived threat, benefits, and barriers in deciding whether or not change behaviour (Carpenter 2010). For participants who felt their sleep could be improved and had negative experiences of disturbed sleep, the threat of continued poor sleep and benefit of sleep improvement led to motivation to engage with REST. For participants who saw neither threat nor benefit, they felt no motivation to change and, in some cases, negative experiences resulted from their beliefs being contradicted.

### 5.2 From Contemplation to Enactment: The Role of Behaviour Change Techniques

A person's ability to move from contemplating change to enacting change is a pivotal if behavioural interventions are to succeed. The behaviour change technique (BCT) taxonomy provides a range of techniques that can be applied to support change (Michie et al. 2013). REST was designed to include BCTs within appointment delivery and the sleep plan. The main BCTs participants found beneficial were information from a credible source, goal setting and action planning, and restructuring of the physical environment.

Information from a credible source took place during the intervention appointment with Extended Scope Practitioners and Advanced Physiotherapists who had experience of working with musculoskeletal and orthopaedic surgery patients. Participants reported high confidence in the health professional's knowledge and advice, which in turn gave them confidence and motivation to engage with the sleep plan (quote 6). Feeling supported and listened to was important. Several participants highlighted that autonomy and having a role in the decision‐making process was valued (quotes 7 and 8).

Goal setting and action planning BCTs were central to REST. Following the assessment, participants were provided with a personalised sleep plan including SMART sleep hygiene goals. This involved agreeing goals which were specific, measureable, achievable, relevant and time‐bound. This process included BCTs for goal setting (behaviour), problem solving, goal setting (outcome), action planning, and discrepancy between current behaviour and goal.

These approaches proved particularly effective for addressing caffeine intake, which was found to be high in many participants. One participant discussed how the appointment helped them become aware of the amount of coffee they were drinking, and to make an achievable plan to reduce this (quotes 9 and 10). Another participant described how having a clear plan for engaging with their chosen ESI (relaxation) helped it feel manageable and achievable (quote 11).

Sleep hygiene recommendations are often centred around another known BCT: restructuring of the physical environment. This focused on creating a positive sleep environment including a bedroom that was dark, cool, comfortable, and free from digital devices, televisions, and pets. The most common change was the removal of the television (quotes 12 and 13). Other participants had added items to the environment such as blackout curtains or low‐level lighting (quotes 14 and 15).

### 5.3 Behavioural Maintenance

Behavioural maintenance is the final stage of supporting long‐term change where the participant is able to sustain the new behaviours over time. Theories of behaviour change maintenance focus on the role of motives, self‐regulation, and habit formation (Kwasnicka et al. 2016).

Self‐regulation theories suggest that motivation to avoid negative consequences, such as adverse effects of poor sleep, is not enough to maintain behaviours that require active effort. To transition into long‐term change, positive reinforcement is needed, such as enjoyment of the activity or positive health results, as highlighted in temporal self‐regulation theory (Hall and Fong 2007) and the model of behaviour maintenance (Rothman 2000). Positive reinforcement leads individuals to psychologically evaluate their behaviour changes as constructive and worth continued investment, and are more likely to make positive self‐judgements about their behaviour, also known as intrinsic motivation (Deci and Ryan 2008; Ryan and Deci 2000).

These theories reflect the experiences of participants who reported improvements in their sleep and were motivated to maintain the change they had made (quotes 16 and 17). One participant talked about making changes to their bedtime routine, which then became habitual and of benefit (quote 18).

Competence and autonomy are core aspects of successful self‐regulation (Ryan and Deci 2000; Ryan et al. 2008). To foster intrinsic motivation individuals need to feel capable of performing the behaviour and that it is their choice to do so. Within behaviour change interventions, this can be supported through shared decision making and tailoring (personalising intervention approaches to meet individual needs). REST offers tailoring through personalised sleep hygiene advice and choice of one of three ESIs. However, data indicated that participants engaged in further tailoring to make their sleep plans work best for them.

Several participants who engaged with mindfulness and relaxation said the recommended resources did not work for them, but had found alternatives. One participant used an alternative mindfulness app (quote 19). Another discussed challenges of using an app in bed when his wife was present, and instead preferred to listen to relaxing music, such as Classic FM (quotes 20 and 21). In these cases, the adaptations proved to be effective and positive for the participants, meaning they could engage in relaxation in ways that suited their lifestyle and preferences.

## 6 Discussion

Improving sleep for patients undergoing TKR can benefit preparation for surgery and post‐surgical recovery. Improving sleep can reduce post‐surgical pain severity and analgesia consumption, support immune function and improve wound healing (Tamrat et al. 2014; Wright et al. 2015). Reduction of pre‐operative pain levels through improved sleep can also benefit patients in the longer term by reducing the risk of developing chronic post‐surgical pain (Sayers et al. 2016).

Behavioural sleep management approaches are dependent on substantial engagement and enactment from individuals, which can be a barrier to success. Exploring engagement and enactment with the REST sleep intervention through health psychology theory has highlighted important areas of consideration in intervention feasibility and future implementation. Health beliefs played a pivotal role in motivation and readiness to change. This was particularly apparent in relation to beliefs about what constituted ‘healthy sleep’ and ‘healthy sleep behaviours’. This reflects previous studies which have indicated that dysfunctional sleep beliefs are maintaining mechanism of insomnia (Thakral et al. 2020). Population level work has found that common dysfunctional beliefs include underestimating the number of hours sleep needed and that it is best to stay in bed if you have trouble sleeping (Pantesco and Kan 2022; Robbins et al. 2019), both of which were identified in this study.

The finding that some participants did not believe they had any issues with their sleep, despite reporting eligible sleep disturbance screening scores, was unexpected but raises important considerations for future iterations of REST and NHS practice implementation. An assessment of sleep beliefs and readiness to change could be added to triage people who may benefit from education on healthy sleep, and raise awareness of how their current sleep behaviours might be impacting their sleep quality and joint pain.

REST was designed as a tailored intervention to support competence and autonomy. Participants appreciated the shared decision‐making process, and the personalised SMART goals. Data highlighted that participants were making further adaptations to the mindfulness and relaxation delivery mechanism. This benefitted engagement levels and supported sustained change; however, it raises potential challenges around intervention fidelity. This is an important assessment at the feasibility stage of intervention development, and is a common tension in interventions which include aspects of tailoring and adaptation (Toomey et al. 2020). Previous explorations of the fidelity/adaptation boundary have suggested that fidelity should be based on causal mechanisms to ensure intervention effectiveness, whilst allowing for a level of adaptation to promote implementation and sustainability (Bopp et al. 2013). Viewed through this perspective, the use of alternative apps for relaxation and mindfulness would be wholey acceptable. The evidence base for music improving sleep quality is less established; however, a recent systematic review indicated effectiveness for sedative music for older adults (Chen et al. 2021). To ensure that adaptations maintain the core causal mechanisms, REST could benefit from providing participants with a wider range of evidence‐based alternatives to select from and include discussions about this selection during the appointment or at the 4‐week follow‐up.

It is important to acknowledge that all participants in REST were English speakers, were able to read and write in English, and were all identified as white British/English or Welsh. Not including a diverse range of participants in the intervention development and feasibility testing limits the transferability of findings and may exacerbate existing health inequalities and access, as the intervention design will not have taken into consideration the views and needs of different groups. Within orthopaedics there are widespread inequalities in the provision of joint replacement surgery, and in outcomes after joint surgery (Klemt et al. 2021; M. C. Smith et al. 2017; Zhang et al. 2016). To ensure that interventions such as REST can benefit all patients, future work should seek to gain involvement from racially marginalised groups in order to understand differences in engagement and relevance, cultural context, and benefit.

## 7 Conclusion

Exploring participants' experiences of engaging in a surgical prehabilitation intervention through the lens of health psychology theory has provided important insights into intervention feasibility, and the stages of behaviour change. This is important for surgeons and multidisciplinary teams to consider when developing new prehabilitation care pathways or when implementing evidence‐based prehabilitation practices. Interview data has highlighted key stages of behaviour change that need to be supported to achieve full benefit from prehabilitation: readiness to change, enactment, and behavioural maintenance. Complex behaviour change interventions which include aspects of tailoring should consider the boundaries of acceptable adaption while maintaining core causal mechanisms, and include methods to explore real‐world implementation and usability during the development process.

## Author Contributions

**Katie Whale:** Study Chief Investigator, conceptualisation (lead), funding acquisition (lead), project administration (lead), methodology (lead), formal analysis (lead), writing – original draft preparation (lead), writing – review and editing (lead). **Emma Johson:** Investigation, data collection (lead), project administration, writing – review and editing. **Rachael Gooberman‐Hill:** conceptualisation, funding acquisition (co‐applicant), methodology, supervision, formal analysis, writing – review and editing.

## Conflicts of Interest

The authors declare no conflicts of interest.

## Data Availability

Data are available on reasonable request. Participants were asked on the consent form if they were willing for their information to be anonymously shared with other researchers to support other research in the future. Anonymised data will be stored on the University of Bristol Research Data Storage Facility (https://data.bris.ac.uk) and will be shared via the University of Bristol Research Data Repository within 6 months of the publication of the study results. Access to the data will be restricted to reasonable requests to ensure that data are only made available to bona fide researchers after a data access agreement has been signed by an institutional signatory.
